# A full-document analysis of the semantic relation between European Public Assessment Reports and EMA guidelines using a BERT language model

**DOI:** 10.1371/journal.pone.0294560

**Published:** 2023-12-15

**Authors:** Erik Bergman, Anna Maria Gerdina Pasmooij, Peter G. M. Mol, Gabriel Westman

**Affiliations:** 1 Swedish Medical Products Agency, Uppsala, Sweden; 2 Dutch Medicines Evaluation Board, Utrecht, The Netherlands; 3 Department of Clinical Pharmacy and Pharmacology, University Medical Center Groningen, University of Groningen, Groningen, The Netherlands; 4 Department of Medical Sciences, Uppsala University, Uppsala, Sweden; Kings College Hospital, UNITED KINGDOM

## Abstract

In the European Union, the Committee for Medicinal Products for Human Use of the European Medicines Agency (EMA) develop guidelines to guide drug development, supporting development of efficacious and safe medicines. A European Public Assessment Report (EPAR) is published for every medicine application that has been granted or refused marketing authorisation within the EU. In this work, we study the use of text embeddings and similarity metrics to investigate the semantic similarity between EPARs and EMA guidelines. All 1024 EPARs for initial marketing authorisations from 2008 to 2022 was compared to the 669 current EMA scientific guidelines. Documents were converted to plain text and split into overlapping chunks, generating 265,757 EPAR and 27,649 guideline text chunks. Using a Sentence BERT language model, the chunks were transformed into embeddings and fed into an in-house piecewise matching algorithm to estimate the full-document semantic distance. In an analysis of the document distance scores and product characteristics using a linear regression model, EPARs of anti-virals for systemic use (ATC code J05) and antihemorrhagic medicines (B02) present with statistically significant lower overall semantic distance to guidelines compared to other therapeutic areas, also when adjusting for product age and EPAR length. In conclusion, we believe our approach provides meaningful insight into the interplay between EMA scientific guidelines and the assessment made during regulatory review, and could potentially be used to answer more specific questions such as which therapeutic areas could benefit from additional regulatory guidance.

## Introduction

In Europe, the Committee for Medicinal Products for Human Use (CHMP) of the European Medicines Agency (EMA) develops guidelines to guide drug development [1]. These EMA guidelines outline expectations with respect to the studies needed to support applicants when preparing marketing authorisation application dossiers for new medicinal products. The ultimate goal is to support development of efficacious and safe medicines, balancing the need for robust high-quality evidence with the need to expedite availability of medicinal products for patients in the EU.

Availability and uptake of EMA guidelines vary with factors such as the therapeutic area and type of active substance in the medicinal product. As the body of available guidelines have expanded over time, older products may have been less informed by formal guidelines at time of approval than more recent products, but this can to some extent have been balanced by other forms of regulatory support during development.

A European Public Assessment Report (EPAR) is published for every medicine application that has been granted or refused marketing authorisation within the EU [2]. The EPAR details the characteristics of the medicinal product, the pharmaceutical, pre-clinical and clinical development programme and includes a comprehensive regulatory assessment of the benefits and risk associated with its use. To our knowledge, the extent to which EMA scientific guidelines are reflected in the European Public Assessment Reports (EPARs) that concludes regulatory assessment at time of marketing authorisation in the EU, has not previously been systematically studied.

As manual analysis of thousands of relatively large documents is very labour-intensive, natural language processing techniques can be used for such tasks. Previously used approaches for semantic full-document matching have often relied on generating one single mathematical representation of the document, including approaches such as TF-IDF and Doc2Vec. However, these approaches each come with their respective disadvantages and cannot capture the full granularity of the content, which may prevent in-detail comparison of partial semantic overlap between documents [3]. Large language models such as BERT provide additional performance and robustness in information retrieval tasks [4]. Since the publication of the original BERT model, several domain-specific models such as PubMedBERT [5] and PharmBERT [6] have been developed for the medical and pharmaceutical domains. However, for information retrieval tasks, a further development of the principles of model training has led to a branch of models called sentence transformers which provide greatly improved performance in task such as similarity search [7, 8].

Here, we describe an algorithm for piecewise matching used together with a sentence BERT language model, allowing full-document semantic comparison to investigate the textual semantic distance between EPARs and EMA scientific guidelines. Further, we investigate which factors are related to this semantic distance with the aim to guide and improve guideline development, implementation, and uptake, both for the European Medicines Regulatory Network and for developers of medicinal products for the European market.

## Materials and methods

### Data collection, curation, and transformation

All currently authorised human medicinal products authorised in a central procedure in the EU between 2008 and 2022 were included. In total, 1024 EPARs for initial marketing authorisations connected to these products were collected from the EMA website on November 18, 2022. Similarly, the latest revision of a total of 669 EMA available scientific guidelines were collected via the EMA recommended search page [9]. Drafts and reflection papers were included, while concept papers and documents not directly providing guidance for medicines development such as presentations, overview of comments and list of participants, were excluded.

All documents were converted to plain text and further split into chunks of 200 words with a 50 word overlap between chunks, except at page breaks where chunk size could be lower than 200 words and chunk overlap could be up to 100 words. A total of 265,757 EPAR and 27,649 guideline text chunks were transformed into a semantic text embedding, a mathematical representation in 768-dimensional space, using the Sentence BERT model (mpnet-all-ver2) [10].

### Full-document semantic matching

#### Piecewise semantic matching

**Fig 1:**
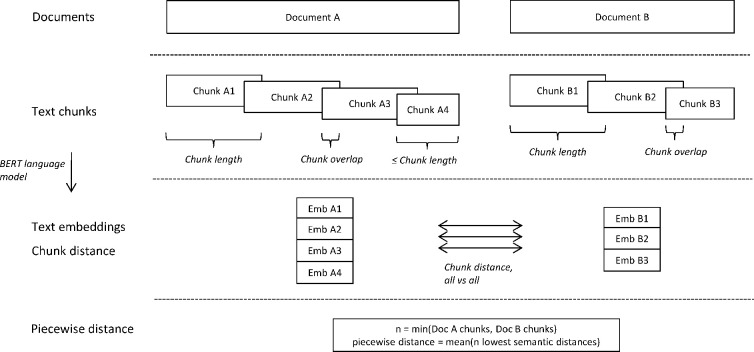
Flow-chart describing the piecewise document matching algorithm. Text chunks from each document are transformed into high-dimensional semantic embeddings using a BERT language model. The embedding vectors are then compared all vs. all between Document A and Document B, whereafter a global document distance measure is calculated based on the geometric mean of the N lowest distances, where N is the number of chunks in the smallest document of the two.

To allow maximum granularity in the analysis of semantic overlap between EPARs and guidelines, an all-vs-all method for piecewise comparison of document chunks was used, generating a set of semantic distances corresponding to the number of chunks in the smallest of the two documents being compared (Fig 1). Hence, a text chunk could contribute more than once if well-matched against multiple chunks in the comparison document. The global semantic distance between two documents was then defined as the mean of the N lowest chunk distances, where N is the number of chunks in the smallest document.

For performance comparison, a mean-pooling algorithm was used, using the mean embedding for each document to calculate the global cosine distance between documents.

#### EPAR vs. guideline semantic analysis

A total guideline semantic distance was calculated for each EPAR, based on the best-matching EMA guidelines. As it was assumed that several, but far from all, guidelines would be applicable to a single regulatory assessment report covering all aspects of drug development for one medicinal product in one therapeutic area, the optimal number of guidelines contributing to the total EPAR semantic distance was investigated. After confirming that using both 1% and 10% of the best guideline matches generated an inferior signal to noise ratio by visual inspection of the expected temporal trend illustrated in Fig 1, this parameter was set to 5% for both piecewise matching and mean pooling algorithms.

*Python 3*.*8* was used for all text and data handling, with libraries; *scikit-learn 1*.*1*.*3*, *cupy-cuda11x 11*.*4*.*0*, *statsmodels 0*.*13*.*5* and *PyMuPDF 1*.*21*.*0*. Factors relating to the guideline semantic distance scores were analysed using linear regression, assuming a statistical significance level of 0.05.

## Results

### EPAR vs. guideline semantic similarity

**Fig 2:**
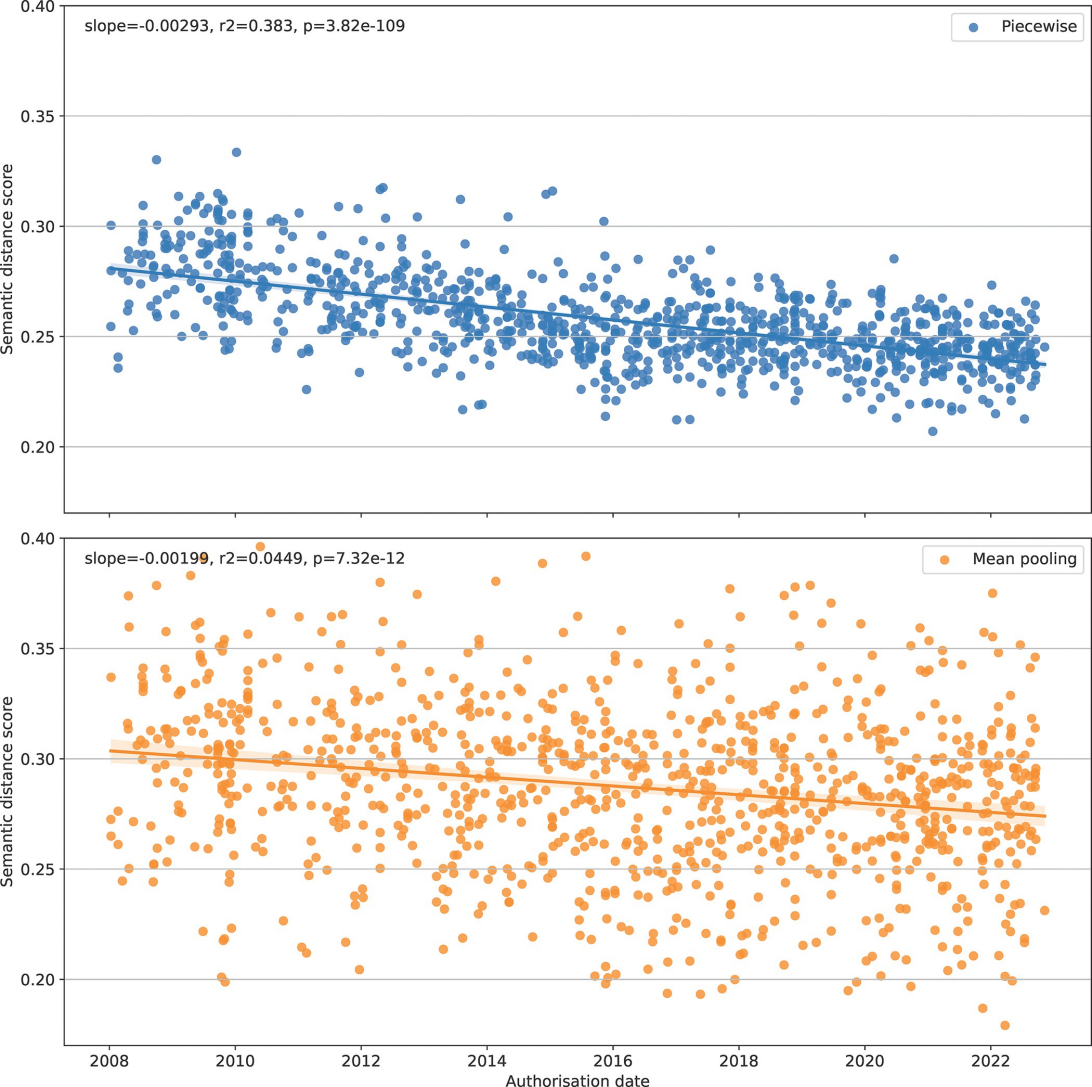
Semantic distance between European Public Assessment Reports for initial marketing authorisation of medicinal products and EMA scientific guidelines, per date of initial marketing authorisation, using piecewise matching (top panel) and mean pooling (bottom panel) algorithms. Lower score indicates higher semantic similarity.

To verify that the scoring algorithms generate meaningful results, the total guideline distance score for each EPAR were compared based on date of approval, with lower score indicating a higher level of semantic similarity (Fig 2). As expected, given that we compare the initial assessment reports with currently available guidelines, the average distance score steadily decreases with time, most clear when using the piecewise matching algorithm (top panel) while the mean pooling (bottom panel) appears to perform less well with a higher variance and a less clear temporal trend.

The piecewise matching method was used to further explore which individual EPARs displayed the lowest semantic distance to EMA guidelines (Table 1). The top-20 list of EPARs with the lowest overall semantic distance to the guidelines include a high proportion of products indicated for the treatment or prevention of Covid-19 (7 products) and Haemophilia A (4 products).

**Table 1:** List of the 20 EPARs with the lowest semantical distance to EMA scientific guidelines. Lower score indicates higher semantic similarity.

Semantic distance	Product name	ATC	MeSH	Date of approval
0.207	Vaxzevria	J07BX03	COVID-19 virus infection	2021-01-29
0.212	Afstyla	B02BD02	Hemophilia A	2017-01-04
0.212	Yargesa	A16AX06	Gaucher Disease	2017-03-22
0.213	Ganirelix Gedeon Richter	H01CC01	Reproductive Techniques, Assisted	2022-07-15
			Ovulation Induction	
			Infertility, Female	
0.213	Veklury		Coronavirus Infections	2020-07-03
0.214	Elocta	B02BD02	Hemophilia A	2015-11-18
0.215	Paxlovid		COVID-19 virus infection	2022-01-28
0.216	Drovelis	G03	Contraceptives, Oral	2021-05-19
0.217	Voncento	B02BD06	Hemophilia A	2013-08-12
			von Willebrand Diseases	
0.217	Dexmedetomidine Accord	N05CM18	Premedication	2020-02-13
0.217	Imcivree	A08AA	Obesity	2021-07-16
0.218	Lydisilka	G03	Contraceptives, Oral	2021-05-19
0.219	NovoEight	B02BD02	Hemophilia A	2013-11-13
0.219	Memantine Accord	N06DX01	Alzheimer Disease	2013-12-03
0.219	Arsenic trioxide medac	L01XX27	Leukemia, Promyelocytic, Acute	2020-09-17
0.220	Jcovden	J07BX03	COVID-19 virus infection	2021-03-11
0.220	Comirnaty	J07BX	COVID-19 virus infection	2020-12-21
0.220	Nuvaxovid	J07BX03	COVID-19 virus infection	2021-12-20
0.220	Spikevax	J07BX03	COVID-19 virus infection	2021-01-06
0.221	Quviviq	N05	Sleep Initiation and Maintenance Disorders	2022-04-29

Inversely, all scientific guidelines were scored to identify those most semantically close to the EPAR database, showing a set of therapeutic indication-independent guidelines with applicability to many medicinal products (Table 2).

**Table 2:** List of the top 20 EMA scientific guidelines contributing to the distance scores, as estimated by mean semantic distance to European Public Assessment Reports for initial marketing authorisation of medicinal products. Lower score indicates higher semantic similarity.

Mean semantic distance	Guideline	Date
0.288	ICH: M 4 Q: Common technical document for the registration of pharmaceuticals for human use—Quality—Step 5	2006-03-08
0.303	Note for guidance on start of shelf-life of the finished dosage form (annex to note for guidance on the manufacture of the finished dosage form)	2001-05-31
0.309	Guideline on the requirements to the chemical and pharmaceutical quality documentation concerning investigational medicinal products in clinical trials—Revision 2	2022-01-27
0.325	Guideline on the non-clinical documentation for mixed marketing authorisation applications—First version	2005-10-13
0.353	Note for guidance on coordinating investigator signature of clinical study reports	2001-10-18
0.378	Note for guidance on in-use stability testing of human medicinal products	2001-03-01
0.4	Note for guidance on the inclusion of appendices to clinical study reports in marketing authorisation applications	2004-06-23
0.43	Guideline on the requirements for quality documentation concerning biological investigational medicinal products in clinical trials—Revision 2	2022-01-27
0.455	ICH M4E (R2) Common technical document for the registration of pharmaceuticals for human use—efficacy—Step 5	2016-07-15
0.491	Guideline on declaration of storage conditions	2007-11-19
0.522	ICH: M 2: Electronic common technical document (e-CTD)—Step 5	2010-09-17
0.54	ICH: M 4 E: Common technical document for the registration of pharmaceuticals for human use—Efficacy—Step 5	2006-03-08
0.541	ICH Topic Q 5 C Quality of biotechnological products: Stability testing of biotechnological/biological products	2006-03-07
0.561	Guideline on safety and efficacy follow-up—risk management of advanced therapy medicinal products	2008-11-20
0.578	Position paper on potential medication errors in the context of benefit-risk balance and risk-minimisation measures	2013-05-30
0.581	CHMP scientific Article-5(3) opinion on the potential risks of carcinogens, mutagens and substances toxic to reproduction when these substances are used as excipients of medicinal products for human use	2007-10-18
0.581	Development pharmaceutics for biotechnological and biological products (CPMP/BWP/328/99) Annex to note for guidance on development pharmaceutics (CPMP/QWP/155/96)	1999-10-21
0.617	ICH guideline Q4B Annex 4C on evaluation and recommendation of pharmacopoeial texts for use in the ICH regions on acceptance criteria for pharmaceutical preparations and substances for pharmaceutical use—Step 5	2017-06-21
0.624	Guideline on the chemistry of active substances	2016-11-15
0.627	ICH guideline E2F on development safety update report—Step 5	2013-02-08

**Fig 3:**
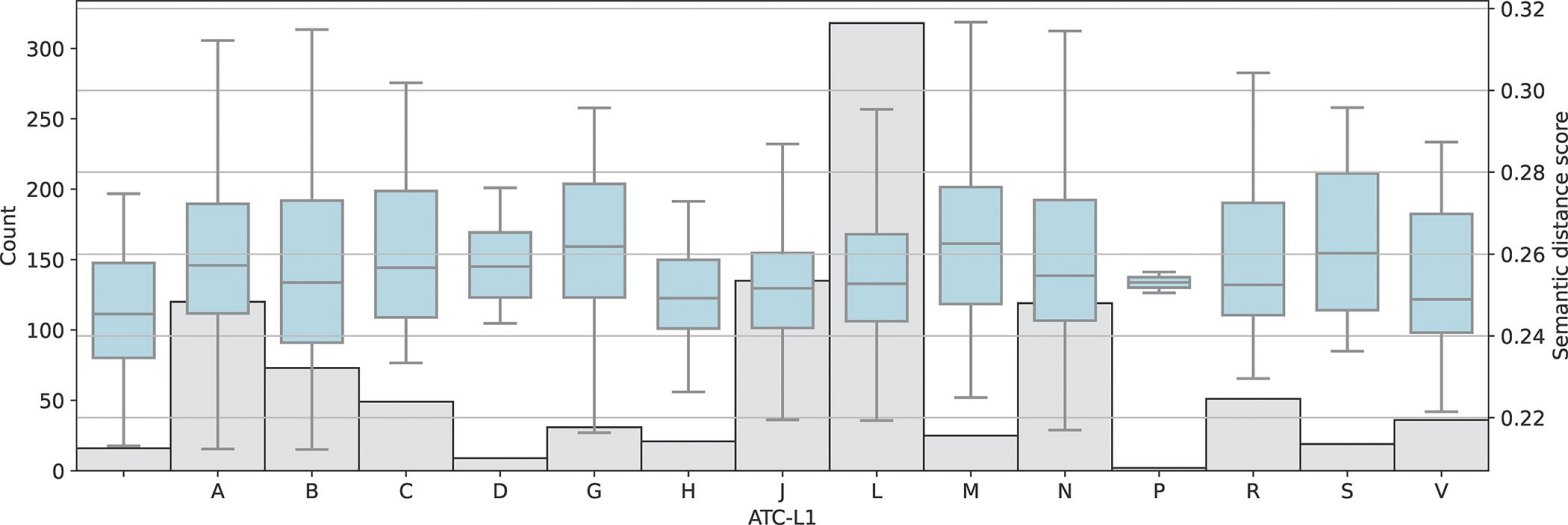
Semantic distance between European Public Assessment Reports (EPARs) for initial marketing authorisation of medicinal products and EMA scientific guidelines per ATC level 1. Number of EPARs in grey. Lower score indicates higher semantic similarity. Some products were yet to be assigned an ATC code at time of analysis.

**Fig 4:**
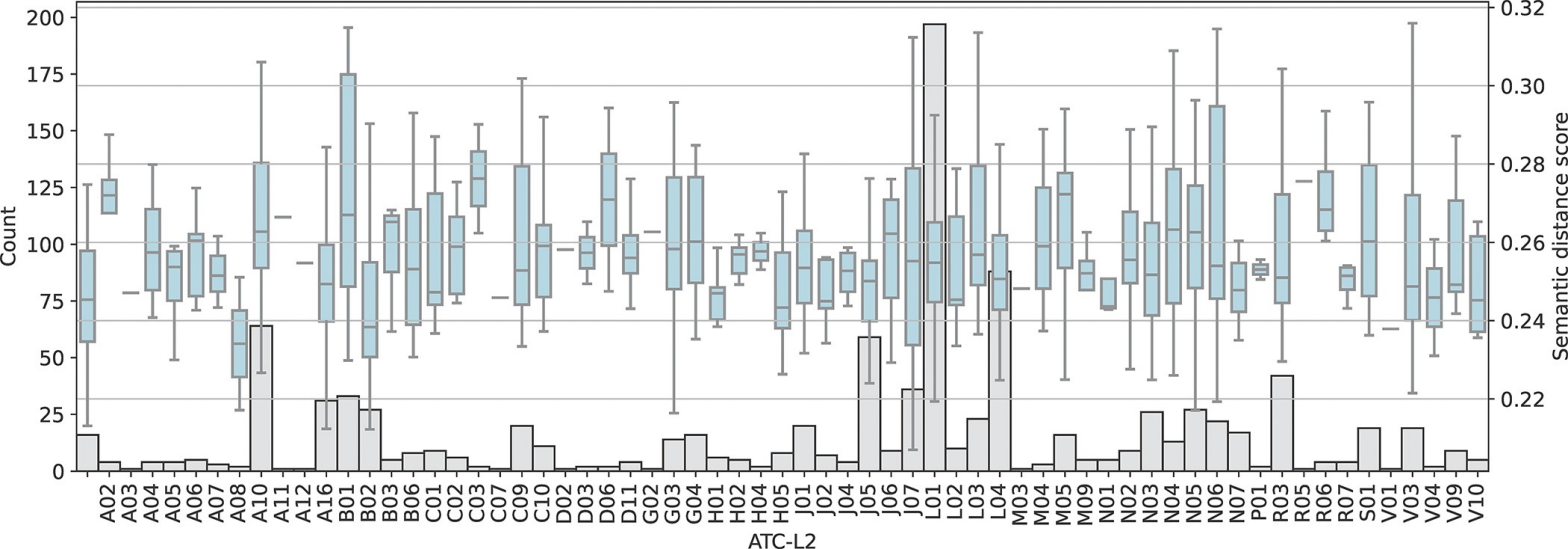
Semantic distance between European Public Assessment Reports (EPARs) for initial marketing authorisation of medicinal products and EMA scientific guidelines per ATC level 2. Number of EPARs in grey. Lower score indicates higher semantic similarity.

**Fig 5:**
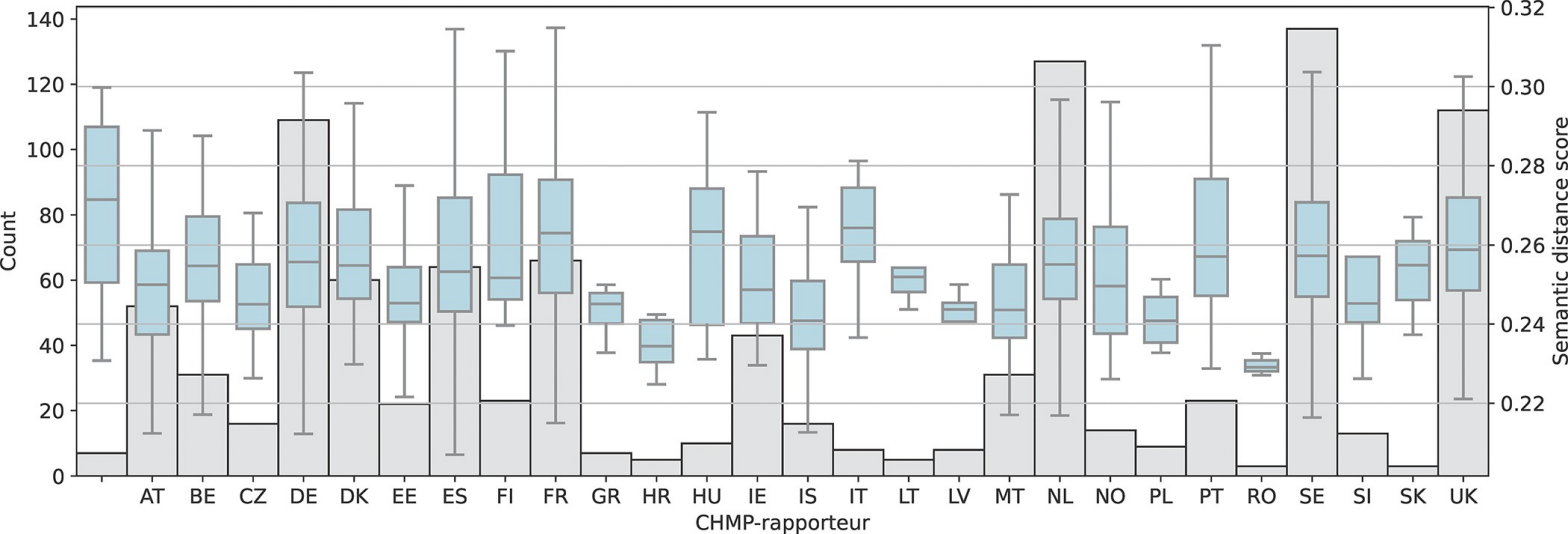
Semantic distance between European Public Assessment Reports (EPARs) for initial marketing authorisation of medicinal products and EMA scientific guidelines per CHMP Rapporteur country. Number of EPARs in grey. Lower score indicates higher semantic similarity.

Factors predicting crude semantic distance to guidelines were investigated, including ATC code levels 1 and 2 (Figs 3 and 4) and CHMP Rapporteur country (Fig 5). The most common ATC level 1 codes in the dataset included antineoplastic agents and immunomodulating agents (L), anti-infectives for systemic use (J), and treatments targeting diseases in the nervous system (N) and related to the alimentary tract and metabolism (A).

Looking at ATC level 2 the differences between product groups were clearer, with anti-obesity agents (A08) and anti-hemorrhagic agents (B02) being the groups with the lowest crude semantic distance to EMA guidelines (excluding group V01 with only one product).

A linear regression model (Table 3 and S1 Table) including product age and document length as covariates was used to further investigate predictors of EPAR-guideline distance in relation to ATC level 2 code, CHMP Rapporteur country and a selected set of metadata variables including whether products were subject to additional monitoring, had orphan designation, were biosimilars, or were subject to a conditional approval.

**Table 3:** Linear regression analyses of EPAR guideline distance by ATC level 2 code, CHMP Rapporteur country and selected metadata parameters.

	Coef.	P-value	CI 95%	
**Product age**	-0.0424	<0.0001	-0.0475	-0.0372
**ATC B02**	-0.0174	<0.0001	-0.0237	-0.0110
**EPAR length**	-0.0177	<0.0001	-0.0255	-0.0100
**Additional monitoring**	0.0045	0.0018	0.0017	0.0073
**ATC J05**	-0.0069	0.0038	-0.0115	-0.0022
**CHMP Rapporteur HR**	-0.0182	0.0108	-0.0321	-0.0042
**CHMP Rapporteur MT**	-0.0084	0.0120	-0.0149	-0.0018
**CHMP Rapporteur LV**	-0.0140	0.0188	-0.0256	-0.0023
**CHMP Rapporteur EE**	-0.0083	0.0282	-0.0157	-0.0009
**CHMP Rapporteur IS**	-0.0092	0.0398	-0.0179	-0.0004

Findings with p-value below 0.05 listed in decreasing order (full model in S1 Table). The largest sub-group for each categorical variable (CHMP Rapporteur country Sweden and ATC L01) were used as contrasting baselines. All variables normalised to the interval 0 to 1 to allow direct comparison of regression coefficients.

EPARs from medicinal products with ATC codes B02 and J05 were associated with a statistically significantly lower semantic distance, indicating a higher textual similarity with guidelines. Similarly, EPARs where the CHMP Rapporteur country was Croatia, Malta, Latvia, Estonia, and Iceland were also significantly more semantically close to guidelines, while reports for products that were flagged for additional monitoring had a higher semantic distance.

The groups with statistically significant differences in semantic distance to guidelines in the linear regression model were explored further, by aggregating distance scores for each group and adjusting for the global geometrical mean distance in the dataset (Table 4 and S2 Table). The results show that the differences observed in relation to ATC code can be attributed to guidelines specific to the therapeutic areas in question. Differences related to the additional monitoring flag appear related to a higher variety of guidelines, and the semantic distance differences are generally lower, but several safety-related guidelines rank high within this group. Further, examples of the best text chunk matches on an individual document level are presented in S1 File.

**Table 4:** List of the top ten EMA scientific guidelines contributing to group-level distance scores for ATC B02, J05 and products under additional monitoring. Scores are adjusted for the respective global geometrical mean in the dataset (see Table 2). Higher score indicates a semantic specificity in relation to the respective group.

Group-specific semantic distance difference	Guideline	Date
**ATC B02—Antihemorrhagics**		
0.586	Guideline on the clinical investigation of recombinant and human plasma-derived factor VIII products—Revision 2	2018-07-26
0.562	Points to consider for assessors. New factor VIII and factor IX products: potency determination for labelling and assays for testing post-infusion samples	2015-11-09
0.561	Draft guideline on clinical investigation of recombinant and human plasma-derived factor VIII products—Revision 2	2017-10-12
0.554	Draft guideline on clinical investigation of recombinant and human plasma-derived factor IX products—Revision 2	2018-11-15
0.552	Guideline on core SmPC for human plasma derived and recombinant coagulation factor VIII products—Revision 3	2018-07-26
0.549	Guideline on the Core SPC for Human Plasma Coagulation Factor VII Products (CPMP/BPWG/2048/01)	2004-07-29
0.545	Draft guideline on core SmPC for human plasma derived and recombinant coagulation factor IX products—Revision 3	2018-11-15
0.526	Guideline on clinical investigation of medicinal products for the treatment of venous thromboembolic disease	2016-02-25
0.505	Questions and answers on the revision of the guidelines on clinical investigation of recombinant and human plasma-derived factor VIII products (EMA/CHMP/BPWP/144533/2009 Rev. 2) and FIX products (EMA/CHMP/BPWP/144552/2009 Rev. 2)	2018-06-08
0.503	Reflection paper on immune-tolerance induction in haemophilia-A patients with inhibitors	2013-03-21
**ATC J05 –Antivirals for systemic use**		
0.461	Guideline on the clinical development of medicinal products for the treatment of HIV infection—Revision 3	2016-04-28
0.297	Elvitegravir/cobicistat/emtricitabine/tenofovir disoproxil film-coated tablets 150 mg/150 mg/200 mg/245 mg product-specific bioequivalence guidance	2017-06-22
0.258	Guideline on the clinical evaluation of medicinal products intended for treatment of Hepatitis B	2006-02-23
0.244	Elvitegravir film-coated tablets 85 mg and 150 mg product-specific bioequivalence guidance	2017-06-22
0.209	Guideline on the clinical evaluation of direct acting antiviral agents intended for treatment of chronic Hepatitis C—First version	2009-04-23
0.193	Emtricitabine/rilpivirine/tenofovir disoproxil film-coated tablets 200 mg/25 mg/245 mg product-specific bioequivalence guidance	2017-06-22
0.141	Guideline on validation of immunoassay for the detection of antibody to human immunodeficiency virus (anti-HIV) in plasma pools	2006-09-21
0.136	Development pharmaceutics for biotechnological and biological products (CPMP/BWP/328/99) Annex to note for guidance on development pharmaceutics (CPMP/QWP/155/96)	1999-10-21
0.125	Guideline on carcinogenicity evaluation of medicinal products for the treatment of HIV infection	2007-12-13
0.125	Reflection paper: Non-clinical and clinical development of similar medicinal products containing recombinant Interferon Alfa	2009-04-23
**Products under additional monitoring**		
0.175	ICH: E 2 F: Development safety update report—Step 3	2008-07-24
0.157	ICH guideline E2F on development safety update report—Step 5	2013-02-08
0.107	Appendix 3 to the guideline on the clinical evaluation of anticancer medicinal products—Summary of Product Characteristics for an Anticancer medicinal product—mock-up of 4.8	2022-01-17
0.105	Note for guidance on the inclusion of appendices to clinical study reports in marketing authorisation applications	2004-06-23
0.105	ICH: E 3: Structure and content of clinical study reports—Step 5	2006-03-08
0.092	ICH: E 1: Population exposure: The extent of population exposure to assess clinical safety—Step 5	2006-03-08
0.088	Guideline on safety and efficacy follow-up—risk management of advanced therapy medicinal products	2008-11-20
0.086	Guideline on manufacture of the finished dosage form—Revision 1	2017-07-04
0.081	Guideline on the chemistry of active substances	2016-11-15
0.075	International Conference on Harmonisation of Technical Requirements for Registration of Pharmaceuticals for Human Use guideline E2C (R2) on periodic benefit-risk evaluation report—Step 5	2013-02-11

## Discussion

To our knowledge, this is the first study that applies a pre-trained language model for semantic analysis of European Public Assessment Reports in relation to EMA scientific guidelines. By using a full piece-by-piece semantic matching method it is possible to capture relevant details across a variety of regulatory document types (S2 File) and directly map sections in the text with high semantic similarity between different documents. This approach can also be used to perform semantic searches that can be applied in regulatory science, using larger sections of text and reconstructing semantic distance with higher complexity than in our previous work on medicinal product information [11].

The piecewise matching method appears to outperform mean pooling of text embeddings in all applications within this study. This is expected due to the preservation of semantic granularity in the process, with the all-vs-all comparison being similar to comparing two puzzles piece-by-piece rather than comparing a single average representation from each puzzle. However, this comes at a price of significantly more computing power required but within the scale of this project, building the full embedding vector database and matching thousands of documents, all calculations were performed in approximately 35 minutes on a single workstation with a consumer-level graphics processing unit (GPU).

Looking into specific results from the linear regression model, it appears that the area of anti-viral drugs (ATC code J05) and antihemorrhagic medicines (B02) have a lower overall guideline semantic distance, also when adjusting for product age and EPAR length. The low semantic distance to guidelines for EPARs in the fields of antivirals appear to be related to active regulatory support for therapeutic areas such as HIV and viral hepatitis. Also, the high number of products in this ATC group provides an increased chance for statistical significance. Similarly, antihemorrhagic medicines are highly standardised, which could be a reason behind the low semantic distance between EPARs in this therapeutic area and current guidelines.

In contrast, EPARs for products that are subject to additional monitoring have an overall higher semantic distance to guidelines when adjusting for product age, which is likely related to the fact that such products more often contain completely new chemical entities or are biological drugs, where guideline support could perhaps be more limited at time of approval due to their novelty [12].

The results related to CHMP rapporteur country is more difficult to interpret, as national agencies have differences in preference for therapeutic areas and types of procedures (i.e., new chemical entities vs. generics applications) and where activity in the European regulatory network has varied over time. Given that a large number of variables were included in the regression model, close to the maximum 1 variable per 10 data points given as a rule of thumb, interactions between variables could not be investigated with statistical rigor.

Importantly, the semantic distance scores presented in this study should not be interpreted as a direct measure of guideline adherence, quality of the dossier provided by the applicant at time of marketing authorisation or quality of the assessment made by the European Regulatory Network. Rather, it is a result of several factors such as the availability of guidelines and level of standardisation in the respective fields.

Nevertheless, we believe that our methodology provides meaningful insight into the interplay between EMA scientific guidelines and the assessment made during regulatory review and could potentially be used to answer more specific questions such as which therapeutic areas could benefit from additional guideline support.

In conclusion, semantic analysis at the whole-document level shows promise in the field of pharmaceutical regulatory science and could potentially assist in identifying therapeutic areas in need of further support by regulatory guidance.

## Supporting information

S1 Table Full linear regression model. (PDF)

S2 Table List of the top ten EMA scientific guidelines contributing to group-level distance scores for the ten EPAR ATC level 2 groups with the most specific guideline matches. Scores adjusted for the respective global geometrical mean in the dataset (see Table 2). Higher score indicates a semantic specificity in relation to the respective group.
(PDF)

S1 File Best product unique text chunk matches between EPARs for medicinal products with ATC B02 and ATC J05 and EMA guidelines. (PDF)

S2 File Multi-document type clustering. (PDF)
