# Supplementary material for: A full-document analysis of the semantic relation between European Public Assessment Reports and EMA guidelines using a BERT language model
Source: PLoS One. 2023 Dec 15;18(12):e0294560. doi: 10.1371/journal.pone.0294560 (PMC10723675; doi:10.1371/journal.pone.0294560)
Supplement: S1 Table — (PDF) [file pone.0294560.s001.pdf]

Supplement 1 – full linear regression model

For categorical variables, the largest subgroups (CHMP Rapporteur country Sweden and ATC L01) were used as contrasting baselines.

	Coef.	Std.Err.	t	P>|t|	CI 95%	
**Constant**	0.2858	0.0021	136.4453	0.0000	0.2817	0.2899
**Product age**	-0.0424	0.0026	-16.1977	0.0000	-0.0475	-0.0372
**ATC B02**	-0.0174	0.0032	-5.3552	0.0000	-0.0237	-0.0110
**EPAR length**	-0.0177	0.0039	-4.4990	0.0000	-0.0255	-0.0100
**Additional monitoring**	0.0045	0.0014	3.1296	0.0018	0.0017	0.0073
**ATC J05**	-0.0069	0.0024	-2.9046	0.0038	-0.0115	-0.0022
**CHMP Rapporteur HR**	-0.0182	0.0071	-2.5549	0.0108	-0.0321	-0.0042
**CHMP Rapporteur MT**	-0.0084	0.0033	-2.5168	0.0120	-0.0149	-0.0018
**CHMP Rapporteur LV**	-0.0140	0.0059	-2.3538	0.0188	-0.0256	-0.0023
**CHMP Rapporteur EE**	-0.0083	0.0038	-2.1975	0.0282	-0.0157	-0.0009
**CHMP Rapporteur IS**	-0.0092	0.0044	-2.0588	0.0398	-0.0179	-0.0004
**ATC A08**	-0.0211	0.0109	-1.9401	0.0527	-0.0425	0.0002
**CHMP Rapporteur IT**	0.0107	0.0057	1.8790	0.0606	-0.0005	0.0220
**ATC A16**	-0.0059	0.0032	-1.8735	0.0613	-0.0122	0.0003
**CHMP Rapporteur CZ**	-0.0079	0.0043	-1.8503	0.0646	-0.0163	0.0005
**ATC C09**	-0.0068	0.0037	-1.8418	0.0658	-0.0141	0.0004
**CHMP Rapporteur RO**	-0.0166	0.0090	-1.8386	0.0663	-0.0343	0.0011
**CHMP Rapporteur UK**	-0.0038	0.0021	-1.8193	0.0692	-0.0078	0.0003
**ATC N03**	-0.0058	0.0033	-1.7483	0.0807	-0.0122	0.0007
**CHMP Rapporteur HU**	-0.0096	0.0058	-1.6553	0.0982	-0.0210	0.0018
**ATC A10**	0.0035	0.0023	1.5153	0.1300	-0.0010	0.0080
**CHMP Rapporteur PL**	-0.0081	0.0056	-1.4544	0.1462	-0.0190	0.0028
**ATC C01**	-0.0075	0.0053	-1.4273	0.1538	-0.0179	0.0028
**ATC A06**	-0.0098	0.0069	-1.4085	0.1593	-0.0234	0.0038
**ATC H05**	-0.0078	0.0056	-1.3901	0.1648	-0.0189	0.0032
**ATC N04**	-0.0062	0.0045	-1.3716	0.1705	-0.0150	0.0027
**ATC D06**	0.0147	0.0108	1.3539	0.1761	-0.0066	0.0360
**ATC G03**	-0.0055	0.0043	-1.3018	0.1933	-0.0139	0.0028
**Orphan designation**	0.0020	0.0017	1.1869	0.2356	-0.0013	0.0054
**ATC C07**	-0.0178	0.0153	-1.1650	0.2443	-0.0477	0.0122
**ATC J04**	-0.0090	0.0080	-1.1358	0.2563	-0.0246	0.0066
**CHMP Rapporteur FI**	0.0041	0.0036	1.1290	0.2592	-0.0030	0.0112
**ATC C02**	-0.0071	0.0064	-1.1153	0.2650	-0.0197	0.0054
**ATC H04**	0.0120	0.0112	1.0772	0.2817	-0.0099	0.0340
**ATC V03**	0.0040	0.0038	1.0667	0.2864	-0.0034	0.0114
**ATC D11**	0.0081	0.0077	1.0483	0.2948	-0.0070	0.0232
**CHMP Rapporteur PT**	0.0037	0.0035	1.0458	0.2959	-0.0032	0.0106
**ATC not assigned**	-0.0042	0.0040	-1.0343	0.3013	-0.0121	0.0037
**ATC N01**	0.0072	0.0070	1.0281	0.3042	-0.0066	0.0211
**ATC B01**	0.0031	0.0031	1.0105	0.3125	-0.0029	0.0091

**ATC S01**	0.0037	0.0037	0.9803	0.3272	-0.0037	0.0110
**ATC N07**	-0.0038	0.0040	-0.9552	0.3397	-0.0116	0.0040
**ATC C10**	0.0045	0.0048	0.9455	0.3447	-0.0049	0.0139
**ATC C03**	0.0101	0.0109	0.9260	0.3547	-0.0113	0.0314
**ATC A05**	-0.0071	0.0078	-0.9179	0.3589	-0.0224	0.0081
**ATC J02**	-0.0055	0.0060	-0.9104	0.3628	-0.0173	0.0063
**ATC V10**	-0.0061	0.0069	-0.8831	0.3774	-0.0197	0.0075
**ATC A11**	-0.0135	0.0153	-0.8797	0.3793	-0.0435	0.0166
**CHMP Rapporteur SK**	0.0078	0.0093	0.8412	0.4005	-0.0104	0.0260
**CHMP Rapporteur NO**	-0.0035	0.0043	-0.8182	0.4135	-0.0120	0.0049
**ATC N02**	0.0044	0.0054	0.8146	0.4155	-0.0062	0.0150
**ATC N05**	-0.0026	0.0032	-0.8038	0.4217	-0.0089	0.0037
**ATC R05**	0.0121	0.0152	0.7924	0.4283	-0.0178	0.0419
**ATC J06**	-0.0042	0.0053	-0.7909	0.4292	-0.0146	0.0062
**CHMP Rapporteur LT**	-0.0058	0.0073	-0.7865	0.4318	-0.0202	0.0086
**CHMP Rapporteur ES**	-0.0019	0.0025	-0.7860	0.4321	-0.0068	0.0029
**ATC R07**	-0.0055	0.0077	-0.7121	0.4766	-0.0206	0.0096
**ATC A12**	0.0108	0.0153	0.7101	0.4778	-0.0191	0.0408
**ATC A04**	-0.0055	0.0079	-0.6958	0.4867	-0.0211	0.0101
**ATC M04**	0.0060	0.0091	0.6663	0.5054	-0.0118	0.0239
**CHMP Rapporteur DK**	0.0016	0.0025	0.6537	0.5135	-0.0032	0.0065
**ATC V01**	-0.0099	0.0152	-0.6505	0.5156	-0.0398	0.0200
**ATC R06**	0.0055	0.0088	0.6215	0.5344	-0.0118	0.0227
**Biosimilar**	0.0015	0.0024	0.6208	0.5349	-0.0032	0.0062
**ATC D02**	-0.0094	0.0153	-0.6159	0.5381	-0.0395	0.0206
**CHMP Rapporteur AT**	-0.0017	0.0027	-0.6120	0.5407	-0.0070	0.0037
**CHMP Rapporteur not assigned**	0.0036	0.0060	0.5983	0.5498	-0.0082	0.0154
**CHMP Rapporteur DE**	0.0012	0.0021	0.5891	0.5560	-0.0029	0.0053
**ATC M05**	0.0023	0.0040	0.5688	0.5696	-0.0056	0.0102
**ATC V09**	0.0030	0.0054	0.5635	0.5732	-0.0075	0.0135
**CHMP Rapporteur BE**	0.0017	0.0033	0.5138	0.6075	-0.0048	0.0081
**ATC H01**	-0.0031	0.0064	-0.4914	0.6233	-0.0157	0.0094
**ATC A07**	-0.0043	0.0089	-0.4861	0.6270	-0.0218	0.0131
**CHMP Rapporteur NL**	0.0009	0.0020	0.4804	0.6310	-0.0029	0.0048
**ATC L03**	-0.0017	0.0036	-0.4645	0.6424	-0.0088	0.0054
**CHMP Rapporteur IE**	0.0013	0.0029	0.4550	0.6492	-0.0044	0.0070
**ATC A03**	-0.0068	0.0153	-0.4447	0.6566	-0.0368	0.0232
**ATC M09**	-0.0029	0.0071	-0.4115	0.6808	-0.0169	0.0110
**ATC V04**	-0.0042	0.0108	-0.3838	0.7012	-0.0254	0.0171
**ATC L04**	-0.0007	0.0020	-0.3386	0.7350	-0.0047	0.0033
**ATC D03**	0.0032	0.0109	0.2903	0.7717	-0.0182	0.0245
**ATC P01**	-0.0031	0.0109	-0.2868	0.7743	-0.0244	0.0182
**ATC J01**	-0.0010	0.0036	-0.2729	0.7850	-0.0081	0.0061
**ATC L02**	0.0014	0.0050	0.2693	0.7877	-0.0085	0.0112
**CHMP Rapporteur GR**	0.0010	0.0061	0.1687	0.8661	-0.0110	0.0131

**ATC N06**	-0.0006	0.0035	-0.1650	0.8689	-0.0075	0.0063
**CHMP Rapporteur SI**	-0.0007	0.0048	-0.1389	0.8895	-0.0101	0.0088
**ATC B03**	-0.0009	0.0069	-0.1279	0.8982	-0.0145	0.0127
**ATC R03**	0.0003	0.0027	0.1264	0.8994	-0.0050	0.0057
**ATC J07**	-0.0003	0.0029	-0.1149	0.9085	-0.0060	0.0053
**ATC H02**	0.0008	0.0069	0.1101	0.9124	-0.0128	0.0143
**ATC G02**	0.0017	0.0154	0.1078	0.9142	-0.0286	0.0319
**ATC G04**	0.0004	0.0041	0.1047	0.9166	-0.0077	0.0085
**Conditional approval**	-0.0003	0.0027	-0.1043	0.9169	-0.0056	0.0051
**CHMP Rapporteur FR**	0.0002	0.0024	0.0818	0.9348	-0.0045	0.0049
**ATC A02**	-0.0006	0.0080	-0.0717	0.9429	-0.0162	0.0151
**ATC M03**	0.0011	0.0153	0.0703	0.9440	-0.0289	0.0310
**ATC B06**	-0.0004	0.0055	-0.0648	0.9484	-0.0112	0.0104
