# Supplementary material for: A full-document analysis of the semantic relation between European Public Assessment Reports and EMA guidelines using a BERT language model
Source: PLoS One. 2023 Dec 15;18(12):e0294560. doi: 10.1371/journal.pone.0294560 (PMC10723675; doi:10.1371/journal.pone.0294560)
Supplement: S2 Table — Scores adjusted for the respective global geometrical mean in the dataset (see Table 2). Higher score indicates a semantic specificity in relation to the respective group. (PDF) [file pone.0294560.s002.pdf]

Supplement 2

List of the top ten EMA scientific guidelines contributing to group-level distance scores for the ten EPAR ATC level 2 groups with the most specific guideline matches. Scores adjusted for the respective global geometrical mean in the dataset. Higher score indicates a semantic specificity in relation to the respective group.

**Group-specific semantic distance difference**	**Guideline**	**Date**
**ATC A02 - Drugs for acid related disorders**		
0.846	Guideline on the evaluation of drugs for the treatment of gastro-oesophageal reflux disease	2011-03-17
0.616	Guideline on quality of oral modified-release products	2014-03-20
0.600	Reflection paper on the dissolution specification for generic solid oral immediate release products with systemic action - First version	2017-08-10
0.529	ICH: M 4 Q: Location issues for common technical document for the registration of pharmaceuticals for human use - Quality questions and answers - Step 5	2006-03-08
0.524	ICH: Q 6 A: Test procedures and acceptance criteria for new drug substances and new drug products: Chemical substances - Step 5	2006-06-28
0.513	Guideline on stability testing: Stability testing of existing active substances and related finished products - Revision 1 (Corr)	2003-12-17
0.509	ICH: Q 1 A (R2): Stability testing of new drug substances and products - Step 5	2006-03-07
0.490	ICH: Q 6 B: Test procedures and acceptance criteria for biotechnological/biological products - Step 5	2006-03-07
0.465	ICH: E 1: Population exposure: The extent of population exposure to assess clinical safety - Step 5	2006-03-08
0.461	Prasugrel hydrochloride film-coated tablets 5 mg and 10 mg product-specific bioequivalence guidance	2018-05-31
**ATC A08 - Antiobesity preparations, excl. diet products**		
0.821	Guideline on clinical evaluation of medicinal products used in weight management - Revision 1	2016-06-23
0.764	Guideline on clinical evaluation of medicinal products used in weight control - Addendum on weight control in children	2008-07-24
0.758	Reflection paper on investigation of pharmacokinetics and pharmacodynamics in the obese population	2018-01-25
0.548	Guideline on stability testing: Stability testing of existing active substances and related finished products - Revision 1 (Corr)	2003-12-17
0.521	ICH guideline Q12 on technical and regulatory considerations for pharmaceutical product lifecycle management - Step 5	2020-03-04
0.520	ICH: Q 1 A (R2): Stability testing of new drug substances and products - Step 5	2006-03-07
0.395	ICH: E 3: Structure and content of clinical study reports - Step 5	2006-03-08
0.390	Guideline on the chemistry of active substances	2016-11-15
0.388	Guideline on stability testing for applications for variations to a marketing authorisation - Revision 2	2014-03-21
0.377	Adopted reflection paper on the use of extrapolation in the development of medicines for paediatrics - Revision 1	2018-10-07
**ATC A06 - Drugs for constipation**		
0.817	Guideline on the evaluation of medicinal products for the treatment of chronic constipation (including opioid induced constipation) and for bowel cleansing	2015-06-25
0.814	Draft guideline on the evaluation of medicinal products for the treatment of chronic constipation	2014-02-20
0.578	Guideline on the clinical development of medicinal products intended for the treatment of pain - First version	2016-12-15
0.384	ICH: E 3: Structure and content of clinical study reports - Step 5	2006-03-08
0.376	Rivaroxaban film-coated tablets 2.5, 10, 15 and 20mg product-specific bioequivalence guidance	2016-04-01

0.324	Guideline on the evaluation of medicinal products for the treatment of irritable bowel syndrome - Revision 1	2014-09-25
0.275	ICH: M 4 E: Common technical document for the registration of pharmaceuticals for human use - Efficacy - Step 5	2006-03-08
0.275	ICH: S 3 B: Pharmacokinetics: Guidance for repeated dose tissue distribution studies - Step 5	2006-03-07
0.274	ICH: M 4 S: Common technical document for the registration of pharmaceuticals for human use - Safety - Step 5	2006-03-08
0.274	Appendix IV of the guideline on the investigation on bioequivalence (CPMP/EWP/QWP/1401/98 Rev.1): Presentation of biopharmaceutical and bioanalytical data in module 2.7.1	2011-11-17
**ATC V10 - Therapeutic radiopharmaceuticals**		
0.780	Guideline on radiopharmaceuticals - Revision 1	2008-11-26
0.773	Guideline on core summary of product characteristics and package leaflet for radiopharmaceuticals	2011-09-23
0.753	Draft guideline on the non-clinical requirements for radiopharmaceuticals - First version	2018-11-15
0.590	Guideline on core summary of product characteristics and package leaflet for fludeoxyglucose (18F)	2012-07-19
0.453	Guideline on core SmPC and Package Leaflet for nanocolloidal technetium (99mTc) albumin - First version	2016-12-15
0.452	Guideline on core SmPC and Package Leaflet for (68Ge/68Ga) generator - First version	2017-07-20
0.445	Guideline on core SmPC and package leaflet for sodium fluoride (18F)	2015-06-25
0.445	Guideline on core core summary of product characteristics (SmPC) and package leaflet for (99Mo/99mTc) generator	2014-12-18
0.438	The use of ionising radiation in the manufacture of medicinal products	1998-12-04
0.434	Guideline on core SmPC and package leaflet for sodium iodide (131I) for therapeutic use	2016-10-13
**ATC R07 - Other respiratory system products**		
0.775	Guideline on the clinical development of medicinal products for the treatment of cystic fibrosis - First version	2009-10-22
0.575	International Conference on Harmonisation of Technical Requirements for Registration of Pharmaceuticals for Human Use considerations (ICH) guideline Q8 (R2) on pharmaceutical development - Step 5	2017-06-22
0.395	ICH: E 3: Structure and content of clinical study reports - Step 5	2006-03-08
0.348	Guideline on control of impurities of pharmacopoeial substances: compliance with the european pharmacopoeia general monograph 'substances for pharmaceutical use' and general chapter 'control of impurities in substance...	2004-04-22
0.330	Guideline on clinical investigation of medicinal products in the treatment of chronic obstructive pulmonary disease	2012-06-21
0.317	CHMP scientific Article-5(3) opinion on the potential risks of carcinogens, mutagens and substances toxic to reproduction when these substances are used as excipients of medicinal products for human use	2007-10-18
0.284	Appendix 3 to the guideline on the clinical evaluation of anticancer medicinal products - Summary of Product Characteristics for an Anticancer medicinal product - mock-up of 4.8	2022-01-17
0.266	Guideline on summary of requirements for active substances in the quality part of the dossier - Revision 1	2005-02-01
0.262	Guideline on risk assessment of medicinal products on human Reproduction and lactation: from data to labelling	2008-07-24
0.243	ICH: S 3 A: Toxicokinetics: A guidance for assessing systemic exposure in toxicology studies - Step 5	2006-03-07
**ATC J04 - Antimycobacterials**		
0.774	Addendum to the guideline on the evaluation of medicinal products indicated for treatment of bacterial infections to address the clinical development of new agents to treat pulmonary disease due to Mycobacterium tubercul...	2017-07-20
0.492	ICH: E 14: The Clinical Evaluation of QT/QTs Interval Prolongation and Proarrhythmic Potential for Non-Antiarrhythmic drugs - Step 5	2006-03-08
0.411	Guideline on manufacture of the finished dosage form - Revision 1	2017-07-04
0.392	International Conference on Harmonisation of Technical Requirements for Registration of Pharmaceuticals for Human Use considerations (ICH) guideline Q8 (R2) on pharmaceutical development - Step 5	2017-06-22
0.364	Guideline on the chemistry of active substances	2016-11-15
0.302	Guideline on the need for non-clinical testing in juvenile animals of pharmaceuticals for paediatric indications	2008-01-24

0.269	Guideline on risk assessment of medicinal products on human Reproduction and lactation: from data to labelling	2008-07-24
0.240	ICH: S 3 A: Toxicokinetics: A guidance for assessing systemic exposure in toxicology studies - Step 5	2006-03-07
0.217	Guideline on repeated dose toxicity - Revision 1	2010-03-18
0.192	Guideline on the use of phthalates as excipients in human medicinal products	2014-11-20
**ATC J07 - Vaccines**		
0.740	Guideline on clinical evaluation of vaccines - Revision 1	2023-01-16
0.736	Questions and answers on the withdrawal of the CPMP Note for guidance on preclinical pharmacological and toxicological testing of vaccines (CPMP/SWP/465)	2016-07-21
0.726	Guideline on quality aspects included in the product information for vaccines for human use - Revision 1	2018-10-18
0.687	Guideline on adjuvants in vaccines for human use (see also explanatory note)	2005-01-20
0.639	Guideline on influenza vaccines prepared from viruses with the potential to cause a pandemic and intended for use outside of the core dossier context	2007-01-24
0.563	Interim guidance on enhanced safety surveillance for seasonal influenza vaccines in the EU	2014-04-10
0.556	Guideline on quality, non-clinical and clinical aspects of live recombinant viral vectored vaccines	2010-06-24
0.533	Note for guidance on the development of vaccinia virus based vaccines against smallpox	2002-06-24
0.532	Influenza vaccines - non-clinical and clinical module	2016-07-21
0.407	Guideline on Influenza vaccines - Quality module Revision 1	2017-07-20
**ATC H04 - Pancreatic hormones**		
0.738	Guideline on non-clinical and clinical development of similar biological medicinal products containing recombinant human insulin and insulin analogues - Revision 1	2015-02-26
0.730	Guideline on clinical investigation of medicinal products in the treatment or prevention of diabetes mellitus - Revision 1	2012-05-14
0.707	Guideline on immunogenicity assessment of therapeutic proteins - Revision 1	2017-05-18
0.580	Guideline on quality documentation for medicinal products when used with a medical device - First version	2021-07-22
0.566	Appendix IV of the guideline on the investigation on bioequivalence (CPMP/EWP/QWP/1401/98 Rev.1): Presentation of biopharmaceutical and bioanalytical data in module 2.7.1	2011-11-17
0.371	ICH: E 3: Structure and content of clinical study reports - Step 5	2006-03-08
0.369	Guideline on stability testing for applications for variations to a marketing authorisation - Revision 2	2014-03-21
0.361	ICH: E 2 F: Development safety update report - Step 3	2008-07-24
0.361	Guideline on the chemistry of active substances	2016-11-15
0.356	Guideline on plastic immediate packaging materials	2005-05-19
**ATC C10 - Lipid modifying agents**		
0.712	Guideline on clinical investigation of medicinal products in the treatment of lipid disorders - Revision 3	2016-06-23
0.532	Paediatric addendum to CHMP guideline on clinical investigation of medicinal products in the treatment of lipid disorders	2012-09-20
0.489	Reflection paper on assessment of cardiovascular safety profile of medicinal products	2016-02-25
0.317	Guideline on the evaluation of medicinal products for cardiovascular disease prevention	2008-09-25
0.241	CHMP scientific Article-5(3) opinion on the potential risks of carcinogens, mutagens and substances toxic to reproduction when these substances are used as excipients of medicinal products for human use	2007-10-18
0.228	ICH M11 guideline, clinical study protocol template and technical specifications - Step 2b	2022-10-26
0.209	Guideline on excipients in the dossier for application for marketing authorisation of a medicinal product - Revision 2	2007-06-19
0.196	ICH: M 2: Electronic common technical document (e-CTD) - Step 5	2010-09-17
0.171	Guideline on clinical investigation of new medicinal products for the treatment of acute coronary syndrome - First version	2017-07-20
0.155	ICH guideline Q4B Annex 4C on evaluation and recommendation of pharmacopoeial texts for use in the ICH regions on acceptance criteria for pharmaceutical preparations and substances for pharmaceutical use - Step 5	2017-06-21

ATC R03 - Drugs for obstructive airway diseases		
0.699	Guideline on clinical investigation of medicinal products in the treatment of chronic obstructive pulmonary disease	2012-06-21
0.668	Guideline on the requirements for clinical documentation for Orally Inhaled Products including the requirements for demonstration of therapeutic equivalence between two inhaled products for use in treatment of asthma and chronic obstructive pulmonary...	2009-01-22
0.626	Guideline on the clinical investigation of medicinal products for the treatment of asthma - Revision 1	2015-10-22
0.606	Guideline for PMS studies for metered dose inhalers with new propellants	2001-11-27
0.554	Replacement of chlorofluorocarbons (CFC) in metered dose inhalation products	1998-12-04
0.490	Guideline on the pharmaceutical quality of inhalation and nasal products	2006-06-21
0.273	Guideline on registry-based studies	2020-09-24
0.267	Guideline on registry-based studies	2021-10-22
0.194	Guideline on equivalence studies for the demonstration of therapeutic equivalence for locally applied, locally acting products in the gastrointestinal tract - Revision 1	2018-10-18
0.189	ICH: E 1: Population exposure: The extent of population exposure to assess clinical safety - Step 5	2006-03-08
