# Supplementary material for: A full-document analysis of the semantic relation between European Public Assessment Reports and EMA guidelines using a BERT language model
Source: PLoS One. 2023 Dec 15;18(12):e0294560. doi: 10.1371/journal.pone.0294560 (PMC10723675; doi:10.1371/journal.pone.0294560)
Supplement: S1 File — (PDF) [file pone.0294560.s003.pdf]

Best text chunk matches

Best product unique EMA guideline semantic matches in EPARs for medicinal products with ATC B02 and ATC J05.

Example 1

Elocta, page 120-121, ATC B02 – Antihemorrhagics

Safety concern	Routine risk minimisation measures	Additional risk minimisation measures
Inhibitor development to FVIII	Section 4.4 of SmPC:<br>Inhibitors<br>The formation of neutralising antibodies (inhibitors) to factor VIII is a known complication in the management of individuals with haemophilia A. These inhibitors are usually IgG immunoglobulins directed against the factor VIII procoagulant activity, which are quantified in Bethesda Units (BU) per mL of plasma using the modified assay. The risk of developing inhibitors is correlated to the exposure to factor VIII, this risk being highest within the first 20 exposure days. Rarely, inhibitors may develop after the first 100 exposure days.<br>Cases of recurrent inhibitor (low titre) have been observed after switching from one factor VIII product to another in previously treated patients with more than 100 exposure days who have a previous history of inhibitor development. Therefore, it is recommended to monitor all patients carefully for inhibitor	No additional risk minimisation measures are proposed.

Assessment report
EMA/671791/2015 Page 120/132

Safety concern	Routine risk minimisation measures	Additional risk minimisation measures
	occurrence following any product switch.<br>In general, all patients treated with coagulation factor VIII products should be carefully monitored for the development of inhibitors by appropriate clinical observations and laboratory tests. If the expected factor VIII activity plasma levels are not attained, or if bleeding is not controlled with an appropriate dose, testing for factor VIII inhibitor presence should be performed.<br>In patients with high levels of inhibitor, factor VIII therapy may not be effective and other therapeutic options should be considered. Management of such patients should be directed by physicians with experience in the care of haemophilia and factor VIII inhibitors.<br>Section 4.8 of SmPC:<br>Patients with haemophilia A may develop neutralising antibodies (inhibitors) to factor VIII. If such inhibitors occur, the condition will manifest itself as an insufficient clinical response.<br>In such cases, it is recommended that a specialised haemophilia centre be contacted.<br>Post Marketing Experience<br>In post-marketing experience […]	

Guideline on the clinical investigation of recombinant and human plasma-derived factor VIII products - Revision 2, page 8

5.3. Immunogenicity

In general, immunogenicity should be investigated prior to marketing authorisation and substantiated with post-marketing studies.

The occurrence of antibodies against factor VIII is a major complication of haemophilia A treatment. The risk of inhibitor occurrence is higher in patients with severe haemophilia A than in patients with moderate and mild disease. In addition the risk may be associated with the number of exposure days the patients received. Patients have the highest risk during the first 50 EDs. Specific product related immunogenicity in plasma products have been observed due to changes in the manufacturing process. Previously treated patients are the most suitable candidates to test the product-related immunogenicity of a factor VIII product as these patients are considered as low risk patients for developing inhibitors. The diagnosis of a factor VIII inhibitor will be based on clinical observations and be confirmed by factor VIII inhibitor testing in the laboratory.

Neutralising antibodies are the most important immunological concern and therefore the following aspects and basic principles should be considered:

• Inhibitor development should be studied in previously treated patients (>150 exposure days, suffering from severe haemophilia A with a factor VIII level < 1%);

• The modified Nijmegen method of the Bethesda assay should be used. Validated testing should be performed in a central laboratory;

• In case of positive results for an inhibitor, an inhibitor retesting using a second separately drawn sample as confirmatory measurement should be performed in a central laboratory. The sampling timepoints should be recorded and included in the SAE report.

• The definitions for thresholds are ≥0.6 BU for “a low titre” inhibitor and >5 BU for a ‘high-titre’ inhibitor.

• Preferably, inhibitor testing should be performed when factor VIII level has reached baseline.

• Conditions influencing factor VIII inhibitor measurements should be screened and documented, like chronic viral infections (e.g. HIV, HCV) or Lupus anticoagulant;

• Detailed patient characteristics should be recorded (e.g. family history, life style, general health status, infection status, type of factor VIII gene mutation, reason for treatment, treatment start date, kind of treatment (on demand, prophylactic, continuous infusion)).

• Recovery should be monitored.

See section 8 Risk Management Plan for further aspects to be considered.

Comment

Both chunks address the topic of the development and monitoring of drug-specific neutralising antibodies, which may inhibit the effect of factor VIII treatment for haemophilia. Assessed as a highly relevant semantic match.

Example 2

Obizur, page 60, ATC B02 – Antihemorrhagics

Response to Obizur Treatment for Subsequent Serious Bleeds
Three subjects experienced a serious bleeding event subsequent to the qualifying bleed that was treated with susoctocog alfa. Bleeding events that were concurrent with or subsequent to the qualifying bleed (i.e. subsequent and non-target bleeds) were controlled in all but 2 subjects who died due to their co-morbidities.
Summary of main efficacy results
The following tables summarise the efficacy results from the main studies supporting the present application. These summaries should be read in conjunction with the discussion on clinical efficacy as well as the benefit risk assessment (see later sections).
Table 18: summary of efficacy for trial OBI-1-301/301a

**Title:** Efficacy and safety of B-domain deleted recombinant porcine factor VIII (OBI-1) in the treatment of acquired haemophilia A due to autoimmune anti-factor VIII inhibitory antibodies			
Study identifier	OBI-1-301/301a		
Design	Open-label, non-randomised, non-controlled multi-centre, multinational		
	Duration of main phase:		Duration for each patient depended on clinical response<br>First subject in: 10th November 2010<br>Last subject out: 9th October 2013
	Duration of Run-in phase:		not applicable
	Duration of Extension phase:		not applicable
Hypothesis	That serious bleeding in subjects with acquired haemophilia will respond to treatment with Obizur within 24hrs of starting treatment.		
Treatments groups	A case series of 28 patients with acquired haemophilia and life and / or limb-threatening bleeding episodes<br>(one additional patient was treated but did not have acquired haemophilia)		
Endpoints and definitions	Primary endpoint	24hr response	the proportion of serious bleeding episodes responsive to Obizur therapy at 24 hours after the initiation of treatment based on assessment of effectiveness and FVIII blood levels<br><br>A positive response was defined as the investigator's assessment that Obizur was effective or partially effective on both the three-point and four-point scales.

Guideline on the clinical investigation of recombinant and human plasma-derived factor VIII products - Revision 2, page 19-20

Annex I –Overview on clinical trial concept

[IMAGE]

19/23

Annex II – Clinical trials with factor VIII products: new products

**Trial, subject**	**Investigation**	**Parameters**
**PTP ≥12y study – pre-authorisation**		
12 haemophilia A patients (PTP ≥12 years; factor VIII <1%) without inhibitors and not actively bleeding	Pharmacokinetics[1]	Incremental recovery, half-life, AUC, clearance.<br><br>Patients should be re-tested after 3-6 months (including factor VIII inhibitor assay).
	Safety	Blood pressure, heart rate, temperature, respiratory rate and adverse events.
5 haemophilia A patients (PTP ≥12 years; factor VIII <1%) undergoing at least 10 surgical procedures	Clinical efficacy	Efficacy of haemostasis, loss of blood and requirement for transfusion. Factor VIII consumption.
	Safety	Adverse events.
Efficacy and safety in 50 PTPs (≥12 years; factor VIII <1% and CD4>200/μl)	Clinical efficacy	Factor VIII consumption, physician's assessment of response in treatment of major bleeds.
	Immunogenicity	Inhibitor titre in Bethesda Units, using the Nijmegen modification of Bethesda assay, immediately before first exposure, ED10-15, ED50-75 and if there is any suspicion of inhibitor development, continue for a minimum of 50 exposure days.
	Safety	Adverse events.

[1] In order to allow for evaluation of a patient's individual response, pharmacokinetic information e.g. existing PK data with the patient's previous factor VIII product (at least historical or recent recovery and half-life) should be available prior to first administration of the factor VIII product.

Comment

Both chunks address the design of clinical trials for medicinal products intended for the treatment of haemophilia. Assessed as a highly relevant semantic match.

Example 3

Voncento, page 89-90, ATC B02 – Antihemorrhagics

In addition, an updated RMP should be submitted: At the request of the European Medicines Agency.
Whenever the risk management system is modified, especially as the result of new information being received that may lead to a significant change to the benefit/risk profile or as the result of an important (pharmacovigilance or risk minimisation) milestone being reached.
Conditions or restrictions with regard to the safe and effective use of the medicinal product to be implemented by the Member States. Not applicable.
Paediatric Data
Furthermore, the CHMP reviewed the available paediatric data of studies subject to the agreed Paediatric Investigation Plan (P/107/2011) and the results of these studies are reflected in the Summary of Product Characteristics (SmPC) and, as appropriate, the Package Leaflet.

Assessment report
EMA/404213/2013 Page 89/91

References
Bray G, Lee M, Buckwalter C, et al. Use of recombinant factor VIII (Recombinate®) in previously untreated patients with haemophilia A. XXI International Congress of the World Federation of Haemophilia, Mexico City 1994; abstract 107.
Committee for Medicinal Products for Human Use (CHMP). Note for Guidance to Assess Efficacy and Safety of Human Plasma-Derived Factor VIII:C and Factor IX:C products in Clinical Trials in Haemophiliacs before and after Authorisation. February 1996, CPMP/198/95.
Committee for Medicinal Products for Human Use (CHMP). Guideline on Core SPC for Human Plasma Derived and Recombinant Coagulation Factor VIII Products - Rev. 1. Draft. 19 July 2007, CPMP/BPWG/1619/1999 rev.1.
Committee for Medicinal Products for Human Use (CHMP). Note for Guidance on the Clinical Investigation of Human Plasma-Derived Factor VIII and IX Products. Draft. 19 October 2000, CPMP/BPWG/198/95 rev.1.
Committee for Medicinal Products for Human Use (CHMP). Guideline on the Clinical Investigation of Human Plasma-Derived Von Willebrand Factor Products. 17 November 2005. CPMP/BPWG/220/02.

Guideline on the clinical investigation of recombinant and human plasma-derived factor VIII products - Revision 2, page 6-7

4. Efficacy: General aspects
Efficacy needs to be demonstrated in clinical trials to be conducted before marketing authorisation combined with the commitment to perform (a) post-authorisation investigation(s) to collect additional clinical data and to bridge in the long-term between the outcome from clinical trials and from routine use. When clinically evaluating human plasma-derived or recombinant coagulation factors for the treatment of haemophilia A, the initial trial typically examines the pharmacokinetics of the principal active factor. Appropriate pharmacokinetic data (incremental recovery, half-life, area under the curve (AUC), and clearance) are the most important surrogate endpoints for efficacy of a new factor VIII product. Furthermore, clinical efficacy of factor VIII treatment (e.g. prophylaxis, on demand) should be assessed during a period of a minimum of 50 exposure days by the patients themselves and treating physicians.

5. Safety: General aspects
Safety aspects of factor VIII products include viral safety, immunogenicity and other adverse events. For recombinant products, the use of non-human cell-lines raises the possibility of different contaminants and altered immunogenic potential.

Guideline on the clinical investigation of recombinant and human plasma-derived factor VIII products
EMA/CHMP/BPWP/144552/2009 rev 2
6/23

Comment

Both chunks address safety and efficacy in the use of factor VIII products for the treatment of haemophilia A. In addition, the reference section in the EPAR contains references to a previous version of a previous EMA guideline on the same therapeutic area. Assessed as a highly relevant semantic match.

Example 4

Trogarzo, page 73, ATC J05 – Antivirals for systemic use

Secondary endpoints	Mean (SD) change in viral load (log10copies/mL)	-1.6 (1.3)	-1.5 (1.4)
	Mean (SD) change in CD4 counts (cells/uL)	36.5 (63.0)	39.8 (80.1)
	Virologic failure or viral rebound	12/59 (20.3%)	13/54 (24.1%)

Analysis performed across trials (pooled analyses and meta-analysis)
The provided comparison of results across studies TMB-301 and TMB-202 suggested that the percentage of patients with a viral load <50 copies/ml at End of Study was similar for the 800 mg Q2W maintenance dose in Study TMB-301 (42.5%) and the 800 mg Q2W group in Study TMB-202 (44.1%). The percentage for the 2000 mg Q4W group, in Study TMB-202, was numerically lower (27.8%) (Table 27). A similar pattern was observed for the proportion of patients with a viral load <400 copies/ml at End of Study.
Table 27 Proportion of Patients with HIV-1 RNA Levels <50 Copies/mL and <400 Copies/mL at End of Study (Study TMB-301 and Study TMB-202, Intent-to- Treat Population)
[IMAGE]
The proportion of patients with a ≥0.5 log10 decrease in viral load from baseline to End of Study was similar for the 800 Q2W maintenance dose in Study TMB-301 (62.5%) and the 800 mg Q2W group in Study TMB-202 (67.8%). The percentage for the 2000 mg Q4W group in Study TMB-202 was 59.3%. The mean change in viral load from baseline to End of Study was -1.5 to -1.64 log10 copies/mL across the three treatment groups in Study TMB-301 and Study TMB-202.

Guideline on the clinical development of medicinal products for the treatment of HIV infection - Revision 3, page 9-10

In all other clinical studies the proportion of patients that achieves and maintains suppression of the plasma viral load to below the limit of quantification (<LLOQ of the HIV-RNA assay used) is the preferred primary efficacy outcome measure. Detectable low level viraemia (i.e. above the LLOQ for those assays with the lowest LLOQ in clinical use, but below a previously applied cut-off such as 50 or 400 copies/mL) could indicate real differences in antiviral potency between regimens. Since future comparative trials are expected to be of non-inferiority designs, the most sensitive virological endpoint possible (i.e. < LLOQ of a suitable assay) should be used. If the 50 copies cut-off is designated as the primary endpoint and used for the sample size calculation, the proportions that achieve and maintain plasma viral loads < LLOQ should also be presented. Viral responses over time should be displayed.
The use of the FDA snapshot algorithm, in which patients with missing values that switch regimens or discontinue assigned treatment are counted as failures, is considered appropriate for the determination of the primary endpoint (2). In addition to presenting the proportion of patients achieving viral loads <LLOQ, the percentages with viral loads falling into pre-defined strata (e.g. 20-49, 50-99, 100-199, 200-400 and > 400 copies/mL) should be tabulated.

Guideline on the clinical development of medicinal products for the treatment of HIV infection
EMEA/CPMP/EWP/633/02 Rev. 3 Page 10/20

There is presently no clinical consensus on when to switch treatment in case of persistence or re-appearance of detectable low level viraemia and such patients are managed on an individual basis. The protocol-defined criteria for changing therapy should be justified in relation to the known qualities of the study drugs (primarily the risk of selecting for resistance to one or more agents within the regimen) and to relevant clinical treatment guidelines.

Comment

Both chunks address virologic endpoints in clinical development of medicinal products for the treatment of HIV. Assessed as a highly relevant semantic match.

Example 5

Rukobia, page 50-51, ATC J05 – Antivirals for systemic use

1. Subjects in the randomised cohort begin open label (OL) dosing on Day 9. Subjects in the non-randomised cohort begin open-label dosing on Day 1.
2. The start of OL FTR 600 mg BID is used as the marker from which all other visits will be measured (i.e. the Week 4 visit for subjects in the randomised cohort will occur 4 weeks after the Day visit; the week 4 visit for subjects in the non-randomised cohort will occur 4 weeks after the Day 1 visit.
3. The study is expected to be conducted until an additional option, a rollover study or marketing approval, is in place.

EMA/702367/2020 Page 51/85

Study Participants
Study participants were heavily treatment-experienced HIV-1 infected male and female subjects ≥18 years of age with a confirmed plasma HIV-1 RNA ≥400 copies/ml and a documented historical or baseline resistance, intolerability and/or contraindications to ARVs in at least 3 classes.
Subjects in the randomised cohort were on their current failing regimen and had at least 1 fully active and available agent in ≤2 ARV classes due to resistance, intolerance or safety considerations whereas subjects with zero remaining fully-active ARVs were assigned to the non-randomised cohort.
While the inclusion criteria defined that full activity of at least 2 drugs from a maximum of two classes should be shown, it was not specified, how many partially active drugs may be available and used for the construction of the OBT. Thus, patients in the randomised cohort had up to 6 ARVs in their initial OBT.
Some non-efficacy OBT changes were allowed during the study that did not impact the Snapshot analysis. These included e.g. changes in one or more ARV in virologically suppressed subjects. Reasons for non-efficacy OBT changes were drug intolerance or adverse events, replacement of a drug due to sourcing issues or addition of TDF for the management of hepatitis co-infection.
Patients with current cardiac abnormalities or a history of cardiac disease were excluded from the study based on the results of the QTc study 206275 showing that a supratherapeutic dose of FTR (2400 mg BID) significantly prolong the QTc interval in healthy adult subjects.

Guideline on the clinical development of medicinal products for the treatment of HIV infection - Revision 3, page 14-15

After the first phase described above, all patients should be treated with the new agent (if appropriate, with randomisation to different doses) in conjunction with an individually optimised background regimen (i.e. in place of the prior failing regimen). The primary efficacy endpoint in this phase is the virological response rates at 24 and 48 weeks (secondary efficacy endpoints), which should be

Guideline on the clinical development of medicinal products for the treatment of HIV infection
EMEA/CPMP/EWP/633/02 Rev. 3 Page 14/20

evaluated according to baseline resistance and activity of OBT. This phase is also important for the assessment of safety through 48 weeks, particularly if a higher dose is used in this population compared to treatment naïve patients (see section 4). These analyses can be used to further understand the durability of the antiviral effect and the need for support from the other agents in the regimen. The longer term outcome achieved with test agent and OBT should be assessed and presented according to genotypic, and if appropriate phenotypic, predicted sensitivity scores (which should be defined) determined at the start of optimized therapy. The applicant should conduct a retrospective exploratory analysis to attempt to identify parameters that are predictive of success, including the need for support from the background regimen of the new agent.
3.4.4. Drug development scenarios not discussed in detail in this guideline

Detailed guidance on other drug development pathways are not provided in this document. For example, specific guidance is not included regarding injectable therapies, NRTI-sparing regimens, induction/maintenance strategies and the conduct of switch studies intended to document not only comparable efficacy but also improved safety.
In all such cases, sponsors should discuss plans for clinical development programmes with EU regulators at intervals.

Comments

Both chunks address clinical trial design for HIV patients with advanced virologic resistance. Specifically, the management of optimised background therapy (OBT) is discussed. Assessed as a highly relevant semantic match.

Example 6

Descovy, page 58-59, ATC J05 – Antivirals for systemic use

Studies Supporting PK and Initial Tolerability in HIV-Infected Subjects (Including PD and PK/PD Relationships)			
GS-US-311-1089<br>PK report for Phase 3 study	**F/TAF** 200/25 mg-tablet + **unboosted 3rd Agent F/TAF** 200/10 mg-tablet + **boosted 3rd Agent**	333	**FTC/TDF** 200/300 mg-tablet + **3rd Agent**
GS-US-120-0104<br>Phase 1	**TAF** 8-mg, 25-mg or 40-mg tablet	25	**TDF** 300-mg tablet
**Studies Evaluating the Effect of Intrinsic Factors**			

Study Number Phase	Test Treatments		Reference Treatment(s) Dose and Formulation
	Dose and Formulation	n	
Studies of the Effect of Renal Impairment			
GS-US-120-0108<br>Phase 1; non-HIV infected subjects	**TAF** 25-mg tablet	27	Not applicable
Studies of the Effect of Hepatic Impairment			
GS-US-120-0114<br>Phase 1; non-HIV infected subjects	**TAF** 25-mg tablet	40	Not applicable
**Studies Evaluating the Effect of Extrinsic Factors**			
Studies of the Effect of Food in Healthy Subjects			
GS-US-311-1386<br>Phase 1	**F/TAF** 200/25-mg tablet	40	Not applicable
Studies of Potential Drug-Drug Interaction in Healthy Subjects			
GS-US-311-0101<br>Phase 1	**F/TAF** 200/40-mg tablet + **EFV** 600-mg tablet<br>**F/TAF** 200/25-mg + 2 × **DRV** 400-mg + **COBI** 150-mg **TAF** 8-mg tablet + **COBI** 150-mg tablet	50	**F/TAF** 200/40-mg tablet<br>**F/TAF** 200/25-mg tablet<br>2 × **DRV** 400-mg + **COBI** 150-mg<br>**TAF** 8-mg tablet
GS-US-342-1167<br>Phase 1	**D/C/F/TAF** F1, 800/150/200/25-mg, monolayer tablet<br>**D/C/F/TAF** F2, 800/150/200/25-mg, bilayer tablet<br>**D/C/F/TAF** F3, 800/150/200/10-mg, monolayer tablet<br>2 × **DRV** 400-mg + **COBI** 150-mg + **FTC/TDF** 200/300-mg tablet	101	2 × **DRV** 400-mg tablet + **COBI** 150-mg tablet<br>**FTC/TDF** 200/300-mg tablet<br>**FTC** 200-mg capsule + **TAF** 25-mg tablet

Guideline on the clinical development of medicinal products for the treatment of HIV infection - Revision 3, page 8

It is not expected that all the drug-drug interaction studies considered to be appropriate or at least desirable will have been performed at the time of initial licensure. In the initial development programme it is recommended that priority should be given to DDI studies with other drugs for the treatment of HIV and for the treatment of concomitant infections (e.g. HCV, HBV, invasive fungal and bacterial infections including mycobacterial diseases), hormonal contraceptives, drugs for the treatment of metabolic abnormalities such as hyperlipidaemia, gastro-oesophageal reflux and drugs used in the management of substance dependence. Within these areas, drugs without reasonable therapeutic alternatives and with a potential for interaction should be prioritized for study. The initial dossier should include a plan for completion of the interaction study programme.

2.4. PK/PD considerations

Data derived from the initial studies in healthy subjects are generally used for the preliminary selection of doses and regimens likely to be effective and tolerable in HIV-infected patients. For example, plasma levels may be compared to protein binding adjusted EC50/95 values for target viruses, to justify target pharmacokinetic indices and the range of doses to be tried in patients with HIV infection.

It is essential that the relationship between drug exposure and safety and efficacy parameters is adequately explored based on data obtained from clinical studies in HIV-infected subjects. Therefore adequate PK sampling should be planned including intensive sampling in subsets of patients. Factors that may impact on drug exposures should be explored by means of population PK analyses (e.g., gender, ethnicity). The results of PK/PD analyses should be taken into account when assessing the potential effects on safety or antiviral efficacy of any alterations in drug exposures that are observed/anticipated in subpopulations (e.g., in patients with hepatic or renal insufficiency, or with potentially significant DDIs).

Comments

Both text chunks address pharmacokinetic (PK) and pharmacodynamic (PD) aspects of clinical trials for medicinal products for the treatment of HIV. Assessed as a highly relevant semantic match.
