# Supplementary material for: A full-document analysis of the semantic relation between European Public Assessment Reports and EMA guidelines using a BERT language model
Source: PLoS One. 2023 Dec 15;18(12):e0294560. doi: 10.1371/journal.pone.0294560 (PMC10723675; doi:10.1371/journal.pone.0294560)
Supplement: S2 File — (PDF) [file pone.0294560.s004.pdf]

Supplement 4 - multi-document type clustering

EPARs for all types of regulatory procedures were included in the clustering. Five EPARs were discarded since the PDF was in graphical format and could not be extracted. EPARs with missing metadata were removed, along with those with duplicated content was also removed.

Guidelines not directly related to the assessment of medicinal products were removed (e.g., presentations, overview of comments, list of participants, etc). Also, concept papers were left out. One guideline was discarded since the PDF was in graphical format and could not be extracted.

A total of 3560 documents (2891 EPARs and 669 guidelines) were used for further analysis.

HDBSCAN (Hierarchical Density-Based Spatial Clustering of Applications with Noise) was used for unsupervised multi-document type clustering based on distance measures from the piecewise and mean pooling algorithms. Clustering was done with default parameters and Euclidean distance for both piecewise and mean pooling distances.

Since no embeddings can be acquired for the piecewise method, a distance matrix was generated directly from the inter-document semantic distances. Clustering generated 73 clusters, of which 11 contained both EPARs and guidelines (Figure FS1). In comparison, the mean pooling embeddings generated 70 clusters with 3 of them containing both EPARs and guidelines. In conclusion, the piecewise distance method generates a higher proportion of multi-document clusters (Figure FS2).

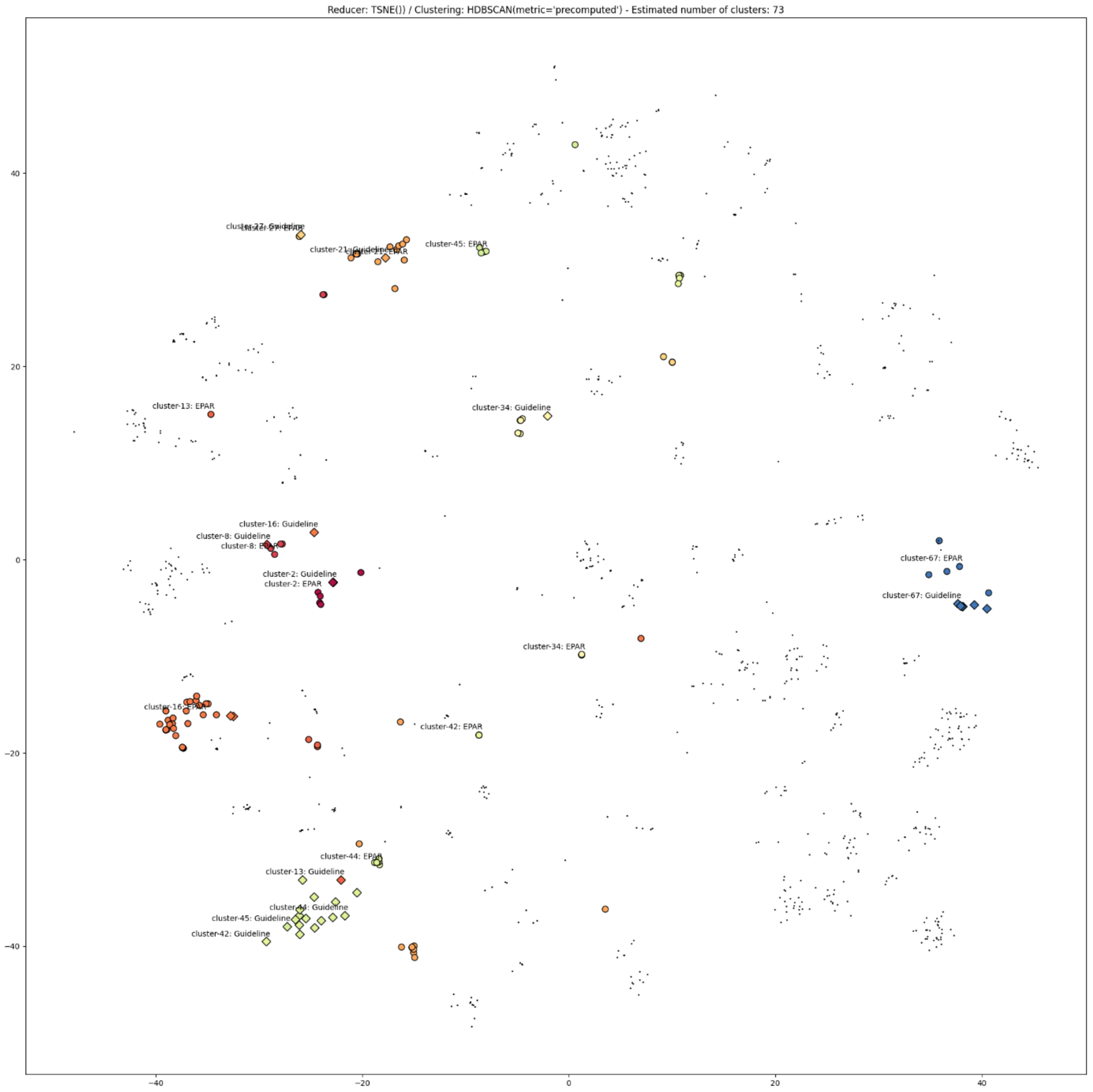

Figure S4-1. t-SNE projection of multi-document type clusters of piecewise distances in colour. Single-document type clusters in black.

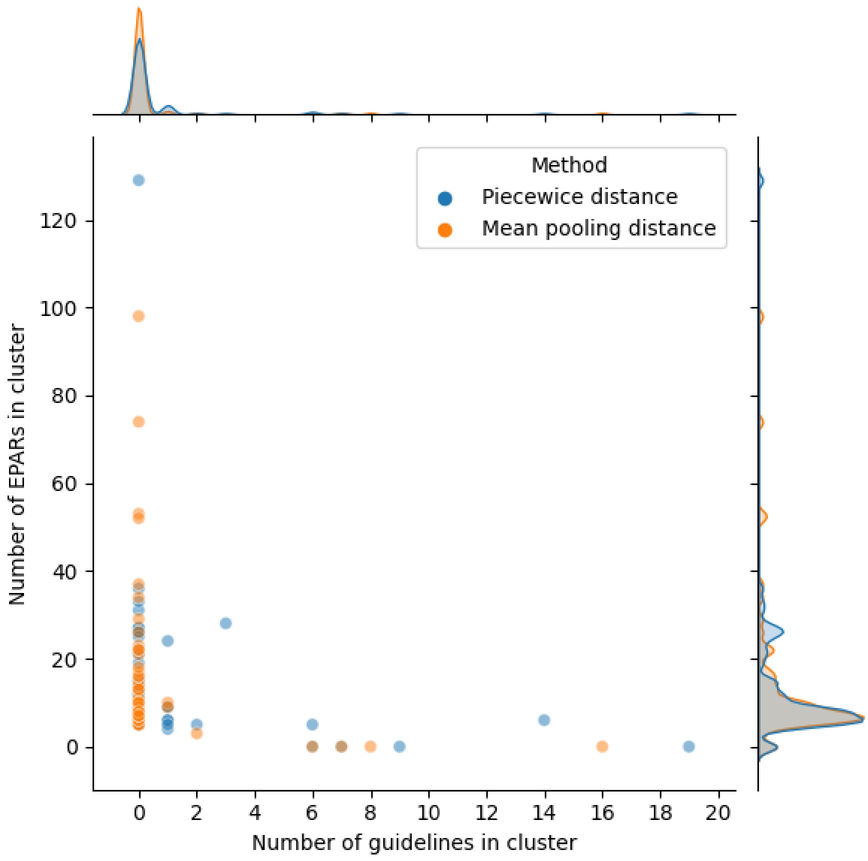

Figure S4-2. Comparison of document clusters generated by the piecewise (blue) and mean pooling (orange) method, based on the number of EPARs and guidelines included in each document cluster.
